# Identification of a New RNA and Protein Integrated Biomarker Panel Associated with Kidney Function Impairment in DKD: Translational Implications

**DOI:** 10.3390/ijms24119412

**Published:** 2023-05-28

**Authors:** Alessandra Scamporrino, Stefania Di Mauro, Agnese Filippello, Grazia Di Marco, Antonino Di Pino, Roberto Scicali, Maurizio Di Marco, Emanuele Martorana, Roberta Malaguarnera, Francesco Purrello, Salvatore Piro

**Affiliations:** 1 Department of Clinical and Experimental Medicine, Internal Medicine, Garibaldi-Nesima Hospital, University of Catania, 95122 Catania, Italy; alessandraska@hotmail.com (A.S.); 8stefaniadimauro6@gmail.com (S.D.M.); agnese.filippello@gmail.com (A.F.); graziadimarco7@gmail.com (G.D.M.); antonino.dipino@unict.it (A.D.P.); robertoscicali@gmail.com (R.S.); maurizio.dimarco@studium.unict.it (M.D.M.); salvatore.piro@unict.it (S.P.); 2 Istituto Oncologico del Mediterraneo, 95029 Viagrande, Italy; emanuele.martorana@grupposamed.com; 3 Faculty of Medicine and Surgery, “Kore” University of Enna, 94100 Enna, Italy; roberta.malaguarnera@unikore.it

**Keywords:** biomarkers, proteins, DKD, RNA, liquid-biopsy

## Abstract

Diabetic kidney disease (DKD) is a complication that strongly increases the risk of end-stage kidney disease and cardiovascular events. The identification of novel, highly sensitive, and specific early biomarkers to identify DKD patients and predict kidney function decline is a pivotal aim of translational medicine. In a previous study, after a high-throughput approach, we identified in 69 diabetic patients 5 serum mitochondrial RNAs (MT-ATP6, MT-ATP8, MT-COX3, MT-ND1, and MT-RNR1) progressively downregulated with increasing eGFR stages. Here, we analyzed the protein serum concentrations of three well-validated biomarkers: TNFRI, TNFRII, and KIM-1. The protein biomarkers were gradually upregulated from G1 to G2 and G3 patients. All protein biomarkers correlated with creatinine, eGFR, and BUN. Performing multilogistic analyses, we found that, with respect to single protein biomarkers, the combination between (I) TNFRI or KIM-1 with each RNA transcript and (II) TNFRII with MT-ATP8, MT-ATP6, MT-COX-3, and MT-ND1 determined an outstanding improvement of the diagnostic performance of G3 versus G2 patient identification, reaching values in most cases above 0.9 or even equal to 1. The improvement of AUC values was also evaluated in normoalbuminuric or microalbuminuric patients considered separately. This study proposes a novel, promising multikind marker panel associated with kidney impairment in DKD.

## 1. Introduction

DKD is a microvascular complication of diabetes that affects approximately 30% of type 1 diabetes (T1D) patients and 40% of type 2 diabetes (T2D) patients [1]. It represents the form of chronic kidney disease attributed to diabetes [2] and is a leading cause of end-stage renal disease (ESRD), responsible for approximately 50% of cases in developed countries. Furthermore, DKD is an important risk factor for cardiovascular disease, and this complication is becoming more prevalent because of the increased incidence of obesity and T2D [3]. DKD is typically diagnosed clinically based on a low estimated glomerular filtration rate (eGFR) and persistently high albuminuria (>300 mg/g creatinine) [4]. Despite their use in clinical practice, eGFR and albuminuria have some notable limitations. Formulas to determine eGFR include serum level creatinine that is influenced by various parameters, including muscular mass, gender, diet, age, and body composition [5]. Furthermore, as far as its prognostic use is concerned, this parameter represents only a late indicator of kidney function decline [6]. Similarly, albuminuria is not specific and has no prognostic value since patients with microalbuminuria can regress to normoalbuminuria [7]. Furthermore, some DKD patients do not show microalbuminuria and maintain a nonalbuminuric phenotype in the course of DKD progression [8,9]. Hence, several studies have been performed to identify diagnostic/prognostic biomarkers of DKD other than albuminuria and eGFR [10,11]. Several novel protein biomarkers have been proposed for DKD diagnosis and progression evaluation, including tumor necrosis factor receptor 1 (TNFRI), tumor necrosis factor receptor 2 (TNFRII), and kidney injury molecule-1 (KIM-1) [11]. 

Tumor necrosis factor-α (TNF-α) is an inflammatory cytokine that has emerged as being critical in the pathogenesis and progression of DKD [12]. TNF-α can act by binding two types of transmembrane receptors: TNFRI and TNFRII. Both receptors can be enzymatically cleaved and realized as soluble forms in the circulation [13]. Several longitudinal studies demonstrated that high circulating TNFR levels are able to strongly predict DKD progression toward ESRD both in proteinuric and nonproteinuric patients [14,15]. Furthermore, these molecules also represent early biomarkers able to predict, in patients with normal renal function, both the fast early eGFR decline and the development of stage 3 chronic kidney disease (CKD-3) both in normoalbuminuric and microalbuminuric patients [16,17]. KIM-1 is a marker of tubular damage. It is expressed on the apical membrane of the proximal tubule cells of the kidney, and in the course of renal injury, its expression level increases [11]. Some studies conducted in diabetic longitudinal cohorts highlighted that circulating KIM-1 levels represent predictors of ESRD, eGFR decline, and progression toward CKD-3 independently of various confounding factors [18,19,20].

As well as protein markers, several studies demonstrated that RNA molecules in body fluids, since they are highly stable and easily detectable [21], can represent biomarkers of diagnosis, prognosis, monitoring, and therapeutic response of several kinds of disease [21,22,23,24]. The use of serum/urine RNA molecules as diagnostic or predictive biomarkers of DKD has been suggested by several studies [22,24,25,26,27]. In a previous study conducted by our research group, after a high-throughput approach, we found that 5 serum novel mitochondrial RNA molecules (MT-ATP8, MT-ATP6, MT-COX3, MT-ND1, and MT-RNR1) in a cohort of 69 T2D patients were progressively downregulated with the increasing eGFR stages (G1: *n* = 24, G2: *n* = 25, G3: *n* = 20) and showed excellent diagnostic performance in G3 versus G2 patient identification. The pathogenesis of DKD is complex and involves different mechanisms, such as metabolic, fibrotic, and inflammatory pathways that crosstalk with each other. Thus, a biomarker panel consisting of several kinds of molecules could be more informative of the various dysregulated molecular mechanisms and could enhance DKD diagnostic/prognostic performance.

In light of this evidence, the main aim of the current study is to determine the serum concentrations of TNFRI, TNFRII, and KIM-1 in the 69 T2D patient cohort and evaluate if their combination with previously identified mitochondrial RNA expression data can constitute a novel panel that allows an enhancement of the diagnostic performance of DKD. 

## 2. Results

### 2.1. TNFRI, TNFRII and KIM-1 Proteins Are Upregulated in DKD

**Figure 1:**
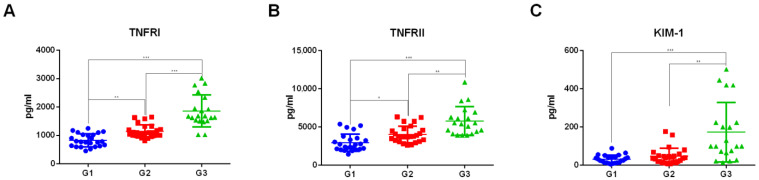
Dot plots of serum levels of TNFRI (**A**), TNFRII (**B**), and KIM-1 (**C**) proteins determined through ELISA assay in patients grouped according to eGFR stages *n* = 69 (G1 = 24, G2 = 25, G3 = 20). Kruskal–Wallis with Dunn’s correction for multiple comparisons was used to evaluate statistical significance. * *p*-value < 0.05. ** *p*-value < 0.01. *** *p*-value < 0.001.

The concentrations of TNFRI, TNFRII, and KIM-1 were determined through ELISA assays in 69 sera samples of diabetic patients grouped according to eGFR stages (stage G1: *n* = 24, eGFR ≥ 90 mL/min/1.73 m^2^; stage G2: *n* = 25, eGFR between 60 and 89 mL/min/1.73 m^2^; stage G3: *n* = 20, eGFR between 30 and 59 mL/min/1.73 m^2^). TNFRI and TNFRII were gradually upregulated, ranging from G1 to G2 and G3 stages. KIM-1 concentration was increased in G3 versus G2 and G3 versus G1 comparisons. For all three protein biomarkers, we observed a strong statistical significance in G3 versus both G1 (*p* < 0.0001) and G2 comparisons (TNFRI *p*-value = 0.0006, TNFRII *p*-value = 0.0076, KIM-1 = 0.0015). The concentrations of the three protein biomarkers are represented as dot plots in Figure 1. Table 1 shows the median concentrations, interquartile ranges, and *p*-values of analyzed protein markers.

**Figure 2:**
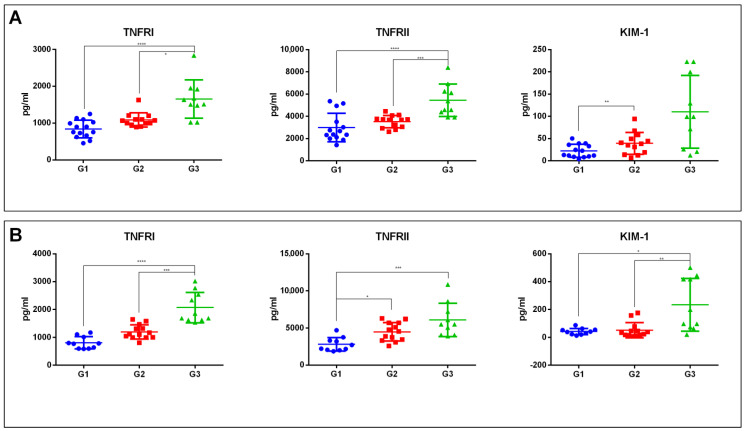
Dot plots of serum levels of TNFRI, TNFRII, and KIM-1 proteins determined through ELISA assay in patients grouped according to eGFR stages in normoalbuminuric *n* = 36 (G1 = 13, G2 = 13, G3 = 10) (**A**) or microalbuminuric patients *n* = 33 (G1 = 11, G2 = 12, G3 = 10) (**B**). Kruskal-Wallis with Dunn’s correction for multiple comparisons was used to evaluate statistical significance. * *p*-value < 0.05. ** *p*-value < 0.01 *** *p*-value < 0.001 **** *p*-value < 0.0001.

We also analyzed if the dysregulations of the three protein biomarkers were maintained in normoalbuminuric or microalbuminuric patients considered separately, thus grouping patients according to the two different parameters, eGFR and ACR: *n* = 36 normoalbuminuric patients (ACR < 30 mg/g) with eGFR stages G1 *n* = 13, G2 *n* = 13, and G3 *n* = 10; *n* = 33 microalbuminuric patients (ACR between 30 and 299 mg/g), with eGFR stage G1 *n* = 11, G2 = 12, and G3 = 10. In normoalbuminuric patients, TNFRI and TNFRII were upregulated in G3 versus both G1 (*p*-value < 0.0001) and G2 patients (TNFRI *p*-value < 0.05, TNFRII *p*-value < 0.001), while KIM-1 was upregulated only in G2 versus G1 comparison (*p*-value < 0.01) (Figure 2A). In microalbuminuric patients, TNFRI and KIM-1 were upregulated in G3 with respect to G1 (TNFRI *p*-value < 0.0001, KIM-1 *p*-value < 0.05) and G2 (TNFRI *p*-value < 0.001, KIM-1 *p*-value < 0.01). The TNFRII levels were increased in G3 versus G1 (*p*-value < 0.001) and G2 versus G1 comparisons (*p*-value < 0.05) (Figure 2B). 

### 2.2. TNFRI, TNFRII and KIM-1 Are Associated with Clinical and RNA Expression Data

In order to test if the analyzed protein markers were associated with both clinical data and previously identified mitochondrial RNA markers, we performed correlation analyses. Since protein marker data followed a nonparametric distribution, Spearman’s correlation was used.

As far as the clinical patients’ data are concerned, in agreement with ELISA assay data, as expected, we found a marked correlation between all the markers and serum creatinine, as well as eGFR (*p*-value < 0.0001). The three proteins also had a strong correlation with blood urea nitrogen (BUN), another marker of renal function. More in detail, TNFRI and TNFRII showed a *p*-value < 0.0001 and KIM-1 a *p*-value of 0.0005. Furthermore, TNFRI, TNFRII, and KIM-1 showed a weak correlation with the albumin/creatinine ratio. Finally, both TNFRI and TNFRII levels were correlated to waist circumference (WC) and hematocrit (HCT). Only TNFRI correlated with AST levels. Table 2 shows the *p*-values of Spearman’s correlation analysis. 

Concerning the correlation between mitochondrial RNA expression data and the analyzed protein markers, we observed that MT-ATP8, MT-ATP6, MT-COX3, and MT-ND1 were associated with TNFRI (*p*-value < 0.006) and TNFRII (*p*-value < 0.04). TNFRI also correlated with MT-RNR1 (*p*-value = 0.0282). KIM-1 levels were associated only with MT-COX3 and MT-ND1 (*p*-value < 0.05) transcripts. Table 3 shows the *p*-values of Spearman’s correlation analysis. 

### 2.3. TNFRI, TNFRII and KIM-1 Show High Performance as Biomarkers of Diabetic Kidney Functional Impairment 

**Figure 3:**
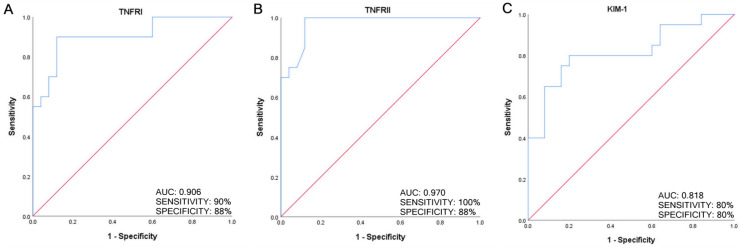
Receiver operating characteristic (ROC) analysis for predicting TNFRI (**A**), TNFRII (**B**), and KIM-1 (**C**) as biomarkers of kidney function impairment in DKD (eGFR stage G3 versus G2).

The diagnostic performance of TNFRI, TNFRII, and KIM-1 in diabetic patients with eGFR stage G3 versus G2 discrimination was analyzed by performing ROC curve analysis. All the protein markers showed excellent diagnostic performance: TNFRI AUC = 0.906, CI = 0.814–0.998, and *p*-value = 4.0 × 10^−6^; TNFRII AUC = 0.970, CI = 0.929–1.000, and *p*-value = 8.0 × 10^−8^; KIM-1 AUC = 0.818, CI = 0.687–0.949, and *p*-value = 2.8 × 10^−4^. Figure 3 shows ROC curves of the three protein biomarkers and reports AUC, sensitivity, and specificity values.

### 2.4. The Combination between Protein Biomarker and Mitochondrial RNA Expression Data Improves the Diagnostic Power for eGFR Stage G3 Patient Identification

To determine if the combination of the analyzed protein biomarkers and RNA expression data could improve the diagnostic performance for G3 patient identification, multivariate logistic analyses were performed. Concerning TNFRI, the combination with each mitochondrial transcript (MT-ATP8, MT-ATP6, MT-COX3, MT-ND1, and MT-RNR1) markedly improved diagnostic performance. The most performant combination were the following: TNFRI + MT-COX3: AUC = 0.964, CI = 0.919–1.000, *p*-value = 4.0 × 10^−6^, sensitivity 85%, and specificity 100%; TNFRI + MT-ATP8: AUC = 0.964, CI = 0.915–1.000, *p*-value = 1.2 × 10^−7^, sensitivity 85%, and specificity 96%. Table 4 reports the AUC, confidence interval, sensitivity, and specificity values of TNFRI alone or in combination with each mitochondrial RNA. 

Regarding TNFRII, except for MT-RNR1, the other mitochondrial transcripts improved diagnostic power. It is important to highlight that the combination of TNFRII with MT-ATP6, MT-COX-3, or MT-ND1 expression levels reached a perfect AUC value (AUC = 1, sensitivity 100%, specificity 100%) (Table 5). 

Finally, as far as KIM-1 is concerned, the combination with all the RNA markers enhanced the diagnostic performance reaching values above 0.9 for MT-ATP8, MT-ATP6, MT-COX3, and MT-ND1. The most performant combination was KIM-1 + MT-ATP6: AUC = 0.926, CI = 0.849–1.000, *p*-value = 1.0 × 10^−6^, sensitivity 95%, and specificity 80% (Table 6). 

### 2.5. The Combination between Protein Biomarker and Mitochondrial RNA Expression Data Improves the Diagnostic Power also in Normoalbuminuric and Microalbuminuric Patients Considered Separately

In order to further characterize our results, we analyzed if the RNA transcript biomarkers were able to improve the diagnostic power of TNFRI, TNFRII, and KIM-1, also considering normoalbuminuric or microalbuminuric patients separately, thus grouping patients according to both of the two different parameters (eGFR and ACR): *n* = 36 normoalbuminuric patients, G1 = 13, G2 = 13, and G3 = 10; *n* = 33 microalbuminuric patients, G1 = 11, G2 = 12, and G3 = 10. More in detail, in these analyses, we took into account MT-ATP8, MT-ATP6, MT-COX3, and MT-ND1, whose dysregulation in G3 versus G2 comparison was maintained considering normoalbuminuric and microalbuminuric patients separately. 

In normoalbuminuric patients, the TNFRI serum levels showed an AUC of 0.869 in G3 versus G2 patient identification. The association between TNFRI and each of the four coding mitochondrial RNA levels strengthened the diagnostic power; indeed, all the AUC values were higher than 0.9. The most efficient combination was given by TNFRI plus MT-ATP-6, which reached an AUC value of 0.992, CI = 0.968–1.000, *p*-value = 7.5 × 10^−5^, sensitivity = 100%, and specificity = 92.3%. Similar results were obtained in microalbuminuric patients. Indeed, the combination with all the RNA markers allowed us to obtain perfect ROC curves with AUC value = 1 (Table 7). 

The best results in normoalbuminuric patients were given by the protein marker TNFRII. In this patient group, the diagnostic performance for G3 patient identification was high, with an AUC value of 0.946. The multivariate logistic analyses performed evaluating TNFRI level together with the expression of the four mitochondrial RNAs demonstrated that the combination of these two kinds of marker determined a perfect ROC curve. The combination of RNA data and TNFRII was not evaluated in microalbuminuric patients since, in this subgroup, TNFRII dysregulation in G3 versus G2 patients did not reach statistical significance (Figure 2B) (Table 8). Hence, the association between TNFRII serum level data with mitochondrial RNA data improves diagnostic power, specifically in normoalbuminuric patients.

Finally, as far as KIM-1 is concerned, the association between KIM-1 and RNA transcripts was evaluated only in microalbuminuric patients, where KIM-1 upregulation in G3 versus G2 was statistically significant. The multilogistic regression analysis with MT-COX3 or MT-ATP8 did not increase the AUC values. The KIM-1 levels showed an AUC of 0.858 in G3 versus G2 comparison; the addition of MT-ND1 (AUC = 0.875) or MT-ATP6 (AUC = 0.867) expression data weakly increased the diagnostic performance (Table 9).

## 3. Discussion

DKD represents a very relevant clinical complication of diabetes. Indeed, it is the main cause of end-stage kidney disease in developed and developing countries and the most common reason for renal replacement therapy [28,29]. Furthermore, this condition is associated with an increase in cardiovascular morbidity and mortality [30]. Thus, preventing the onset of DKD and its progression to ESRD is important. To date, eGFR and albuminuria are the two main indicators commonly used in clinical practice to evaluate the presence and progression of diabetic nephropathy [31]. However, both of them show some notable limitations. These measures are not specific, do not directly determine renal tissue damage, and do not reflect relatively small changes in renal function [31]. For all these reasons, the identification and validation of novel noninvasive diagnostic and prognostic biomarkers for DKD have been the focus of extensive research. Several studies have identified DKD diagnostic and prognostic biomarkers using a pathophysiological pathway-based approach. These identified biomarkers include, for instance, TNFRI and TNFRII, which are involved in the inflammatory TNFα pathway [13] and KIM-1, biomarkers of proximal tubular damage [32]. In this study, we determined the serum concentration of these protein markers through the ELISA assay in a cross-sectional cohort of 69 diabetic patients grouped according to eGFR stages (*n* = 24 G1, *n* = 25 G2, *n* = 20 G3), independently or dependently of ACR stages. As expected, we observed a gradually increasing trend of TNFRI, TNFRII, and KIM-1 concentrations ranging from G1 to G2 and G3 stages. In agreement with these data, all three protein markers correlated with serum creatinine, as well as eGFR. The three proteins also had a strong correlation with BUN, another marker of renal function. Although these protein biomarkers have been mainly analyzed in longitudinal cohorts, some studies with cross-sectional design are in agreement with our data. For instance, Monika A Niewczas et al. observed that elevated concentrations of serum TNFRI and TNFRII levels are strongly associated with decreased renal function in nonproteinuric T1D patients [33]. J Lin et al., in a cross-sectional study of 732 men with T2D, demonstrated that TNFRII levels were significantly higher in subjects with GFR < 60 mL/min/1.73 m^2^ with respect to GFR ≥ 90 mL/min/1.73 m^2^, and through multivariable logistic regression analysis they demonstrated that TNFRII together with triglycerides, fibrinogen, and VCAM were associated with increased odds of having a GFR < 60 mL/min/1.73 [34]. Tomohito Gohda et al., in a cross-sectional study that included 499 patients with T2D, observed that TNFRI and TNFRII were negatively associated with the eGFR, and after adjustment for relevant covariates, the serum TNFRs were associated with a lower eGFR (60–89 mL/min/1.73 m^2^) and an increased ACR (≥30 mg/gCr) [35]. In another study in a cohort of 76 patients with T2DM divided into 2 groups according to eGFR (> or <60 mL/min/1.73 m^2^), TNFRI levels were higher in patients with renal alteration function [36].

In a previous study conducted by our research group, we applied a high throughput transcriptomic approach to discover novel biomarkers in a nonbiased manner. After microarray analysis, conducted in 12 serum samples (*n* = 6 with DKD, *n* = 6 without DKD), 33 downregulated transcripts were identified. Among them, by real-time PCR, the downregulation of 5 mitochondrial RNAs (MT-ATP6, MT-ATP8, MT-COX3, MT-ND1, and MT-RNR1) was validated in the same cohort of 69 patients stratified according to eGFR stage. This was the first study that highlighted the differential expression of mitochondrial RNAs in DKD. Other studies, most of them conducted in cross-sectional cohorts, reported several microRNAs dysregulated associated with ACR and/or eGFR [37]. These microRNAs were not considered in the multikind marker panel in order to increase their AUC performance. Although in our previous study, each identified mitochondrial RNA biomarker showed high diagnostic performance, multivariate logistic analysis, performed by combining the expression data of all the identified transcripts and each possible combination of them, did not improve the identified biomarker diagnostic performance because of the problematic amount of collinearity and redundancy of the transcript expression data [38]. For this reason, here, we attempted to combine previously identified mitochondrial RNA expression data with the serum protein expression of the well-validated biomarkers of DKD: TNFRI, TNFRII, and KIM-1. The multivariate logistic analysis showed encouraging results. Grouping patients according to eGFR stages regardless of ACR stage, we found that the combination between (I) TNFRI or KIM-1 with each RNA transcript, (II) TNFRII with MT-ATP8, MT-ATP6, MT-COX-3, and MT-ND1 determined an outstanding improvement of the diagnostic performance of G3 versus G2 patient identification, reaching values in most cases above 0.9 or even equal to 1. 

Through the analyses conducted in the current study, we observed that the approach of combining different kinds of biomarkers (circulating RNAs and proteins) involved in different pathways (mitochondrial dysfunction, inflammation, and tubular damage) strongly increased the diagnostic power for the identification of diabetic patients with functional kidney impairment. This is not surprising; indeed, the development and progression of DKD involve complex multiple pathogenic pathways such as inflammation, fibrosis, oxidative stress, and hemodynamic [39]; hence, a single biomarker could not describe the overall disease progression process and reach excellent diagnostic and prognostic power [40]. On the contrary, a biomarker panel including several kinds of biomolecules involved in different aspects of renal dysfunction and damage could reflect the patient’s actual pathophysiological status more accurately and thus improve the diagnosis and progression management of DKD. 

Some very robust studies conducted in wide longitudinal cohorts simultaneously analyzed several protein and biochemical biomarkers specific to different pathogenic processes to establish a model that is able to improve the predictive performance toward renal function decline. In the study funded by the SUMMIT consortium, within a broad set of 207 serum protein and metabolite biomarkers measured in 154 incident cases of rapid progression of renal function decline and 153 nonprogressing, 14 biomarkers determined an improvement of the predictive performance beyond clinical covariates [41]. In the study funded by the SYSKID consortium, conducted in a longitudinal cohort of 82 patients, from among 28 biomarkers, a panel of 9 protein biomarkers involved in fibrosis, angiogenesis inflammation, and endothelial function improved the prediction of accelerated eGFR decline [42]. In the CACT1 study, the addition of β-2 microglobulin, cystatin C, neutrophil gelatinase-associated lipocalin, and osteopontin to traditional risk factors, significantly improved C-statistics and net-reclassification for incident-impaired eGFR [43]. In another more recent study, in a longitudinal cohort of 1538 patients where changes in eGFR over time and the development of a composite kidney outcome (CKD incidence, CKD progression, or end-stage renal disease) have been evaluated, a multimarker score based on the detection of three urinary proteins MCP-1, YKL-40, and UMOD levels increased prognostic accuracy by improving the AUC and the net-reclassification [44].

The major limitations of our study are determined by the small sample size and the cross-sectional study design, which do not allow us to determine the progression predictive value of identified biomarker panel. However, to the best of our knowledge, this is the first study that combines data on serum protein and RNA molecule expression to establish a novel panel of biomarkers associated with kidney function impairment. These data should be validated in a larger independent external cohort. Furthermore, in order to determine if the identified panel can have a prognostic and predictive value, the model performance should be assessed in association with kidney function decline in longitudinal cohorts. Even with these limitations, this study suggests a novel outstanding performant unexplored biomarker panel for DKD.

## 4. Materials and Methods

### 4.1. Study Population

Sixty-nine diabetic patients were recruited by the Internal Medicine Unit of the Garibaldi-Nesima Hospital, University of Catania, Italy. Diabetes diagnosis was established according to the following parameters: fasting blood glucose ≥ 126 mg/dL and HbA1c ≥ 6.5% (48 mmol/mol) [45]. Albumin/creatinine ratio (ACR) and eGFR were determined to evaluate the presence of DKD complications. The study population was stratified based on eGFR stages (stage G1: *n* = 24, eGFR ≥ 90 mL/min/1.73 m^2^; stage G2: *n* = 25, eGFR between 60 and 89 mL/min/1.73 m^2^; stage G3: *n* = 20, eGFR between 30 and 59 mL/min/1.73 m^2^). We excluded from our study patients with T1D, nondiabetic kidney disease, hepatic diseases, autoimmune disorders, cancer, and diabetes complicated with cardiovascular diseases. The biochemical/clinical data of enrolled subjects are reported in Table S1. There was no significant difference between the studied groups, except for levels of uric acid, blood urea nitrogen, creatinine, HCT, systolic blood pressure, diastolic blood pressure, and presence of hypertension condition (one-way ANOVA or Kruskal–Wallis test *p*-value < 0.05). Detailed pharmacological data of subjects grouped according to eGFR or ACR are reported in Tables S2 and S3, respectively. It is important to highlight that for (ACEi/ARBs) and SGLT2 inhibitor therapies, there were no statistical differences in subjects grouped according to ACR or eGFR values. The same study population had already been used in our previous work, where we identified novel mitochondrial RNA biomarkers associated with kidney functional impairment (progressively downregulated ranging from G1 to G2 and G3) [38]. 

### 4.2. Sample Processing

After one hour at room temperature, blood samples were centrifuged at 3500 rpm at 4 °C for 15 min to separate from whole blood. The serum was further centrifuged to remove eventual cell debris. The upper-layer supernatant was collected, and aliquots were stored at −80 °C until analysis [23].

### 4.3. RNA Analysis

Total RNA extraction was performed from 400 μL of serum by using the miRNeasy mini kit (QIAGEN) according to the manufacturer’s instructions. RNA concentration and quality were determined through the NanoDrop One (Thermo Fisher Scientific, Waltham, MA, USA). The expression of MT-ATP8, MT-ATP6, MT-COX3, MT-ND1, and MT-RNR1 in the 69 sera of diabetic patients was determined through real-time PCR (Power SYBR Green RNA-to-CT1-Step Kit (Thermo Fisher Scientific) in a QuantStudio 5 system (Thermo Fisher Scientific). ACTB was used as a reference gene, and relative quantification data were determined through the 2−ΔΔCt method. Primer sequences were determined by using Primer Blast. MT-ATP8: forward ACAGTGAAATGCCCCAACTAAAT reverse AGGGAGGTAGGTGGTAGTTTGTG; MT-ATP6: forward ACCTTCCCTCTACACTTATCATCTT reverse CGTGCAGGTAGAGGCTTACT; MT-COX3 forward TTCACCATTTCCGACGGCAT reverse GGCGGATGAAGCAGATAGTGA; MTND1 forward CGGGCTACTACAACCCTTCG reverse AGATGTGGCGGGTTTTAGGG; MTRNR1 forward GGGTTGGTCAATTTCGTGCC reverse ACACTCTTTACGCCGGTTTCT; ACTB2 forward GAGCACAGAGCCTCGCCTTT reverse GAGCGCGGCGATATCATCA [38].

### 4.4. ELISA Assays

The concentrations of TNFRI, TNFRII, and KIM-1 in the 69 sera of diabetic patients were determined through ELISA assays (R&D systems, Minneapolis, MN, USA) according to the manufacturer’s instructions. Preliminary experiments were performed to determine the most appropriate dilution factor for each specific assay. Hence, serum was diluted at 1:35 and 1:20, respectively, for TNF-RI and TNF-RII, while dilution was not necessary for the KIM-1 assay. Concentrations were determined by interpolating data from a standard curve generating a four-parametric logistic (4-PL) curve fit as reported in the manufacturer’s instructions.

### 4.5. Statistical Analysis

To assess if clinical and ELISA assay data followed a parametric or nonparametric distribution, three different normality tests were used: the D’Agostino-Pearson omnibus test, the Shapiro–Wilk normality test, and the Kolmogorov–Smirnov normality test [46]. The one-way ANOVA or Kruskal–Wallis test was used to analyze the statistical significance of expression results for normal or not normal data in patients with increasing eGFR stages (G1, G2, and G3).

In order to establish if TNFRI, TNFRII, and KIM-1 serum levels were associated with both transcript expression values and subject clinical data, Spearman’s correlation analyses were performed. The statistical analyses were performed using GraphPad Prism 6.0 (GraphPad Software, Inc., San Diego, CA, USA) [47,48]. ROC curve analysis and multivariate logistic analyses were performed using SPSS Statistics V.27 [22].

## 5. Conclusions

This study demonstrated that the simultaneous dosage of serum mitochondrial RNAs and TNFRI, TNFRII, and KIM-1 proteins can effectively strongly improve diagnostic performance for the evaluation of kidney function impairment in the context of DKD. In general, we propose a multikind marker panel consisting of molecules characterized by different biochemical natures (RNAs plus proteins) associated with kidney impairment in DKD. This approach can be applied to every aspect of translational medicine. 

## Figures and Tables

**Table 1:** Serum concentrations of TNFRI, TNFRII, and KIM-1 reported as median value and interquartile ranges in patients grouped according to eGFR stages *n* = 69 (G1 = 24, G2 = 25, G3 = 20). Kruskal–Wallis with Dunn’s correction for multiple comparisons was used to evaluate statistical significance.

	G1	G2	G3	G1 vs. G2	G1 vs. G3	G2 vs. G3
TNFRI (pg/mL)	777.7 (627.1–1022)	1143 (990.1–1236)	1681 (1517–2238)	0.0089	<0.0001	0.0006
TNFRII (pg/mL)	2526 (2083–3462)	3731 (3189–4610)	5441 (4150–6752)	0.0120	<0.0001	0.0076
KIM-1 (pg/mL)	27.59 (12.27–41.16)	36.92 (17.10–51.82)	99.00 (66.31–222.9)	0.9636	<0.0001	0.0015

**Table 2:** *p*-values of Spearman’s correlation analysis between TNFRI, TNFRII, and KIM-1 serum levels and clinical data. WC: waist circumference; ALT: alanine transaminase BUN: blood urea nitrogen; ACR: albumin creatine ratio; HCT: hematocrit.

	TNFRI	TNFRII	KIM-1
WC (cm)	0.0028	0.0044	0.2433
ALT (UI/L)	0.0313	0.2877	0.6637
Uric Acid (mg/dL)	0.0084	0.0057	0.0079
Creatinine(mg/dL)	<0.0001	<0.0001	<0.0001
BUN (mg/dL)	<0.0001	<0.0001	0.0005
ACR	0.0416	0.0234	0.0494
eGFR CKD-EPI (mL/min/1.73m2)	<0.0001	<0.0001	<0.0001
HCT (%)	0.0015	0.0002	0.0673

**Table 3:** *p*-values of Spearman’s correlation analysis between TNFRI, TNFRII, and KIM-1 serum levels and mitochondrial RNA expression data.

	TNFRI	TNFRII	KIM-1
MT-COX3	0.0055	0.0373	0.0492
MT-ND1	0.0053	0.0273	0.0433
MT-ATP8	0.0046	0.0173	0.1190
MT-ATP6	0.0053	0.0287	0.1040
MT-RNR1	0.0282	0.0560	0.1907

**Table 4:** Results of receiver operating characteristic (ROC) curve and multivariate logistic analyses for predicting, respectively, TNFRI alone or in combination with specific mitochondrial RNAs (MT-COX3, MT-ND1, MT-ATP8, MT-ATP6, or MT-RNR1) as biomarkers of kidney function impairment in DKD (eGFR stage G3 versus G2). *n* = 69 (G1 = 24, G2 = 25, G3 = 20). AUC = area under the curve. CI = confidence intervals.

	AUC	CI	*p*-Value	Sensitivity	Specificity
TNFRI	0.906	(0.814–0.998)	4.0 × 10^−6^	90%	88%
TNFRI + MT-COX3	0.964	(0.919–1.000)	1.2 × 10^−7^	85%	100%
TNFRI + MT-ND1	0.954	(0.899–1.000)	2.2 × 10^−7^	85%	96%
TNFRI + MT-ATP8	0.964	(0.915–1.000)	1.2 × 10^−7^	85%	100%
TNFRI + MT-ATP6	0.962	(0.915–1.000)	1.3 × 10^−7^	85%	96%
TNFRI + MT-RNR1	0.950	(0.893–1.000)	2.7 × 10^−7^	90%	88%

**Table 5:** Results of receiver operating characteristic (ROC) curve and multivariate logistic analyses for predicting, respectively, TNFRII alone or in combination with specific mitochondrial RNAs (MT-COX3, MT-ND1, MT-ATP8, MT-ATP6, or MT-RNR1) as biomarkers of kidney function impairment in DKD (eGFR stage G3 versus G2). *n* = 69 (G1 = 24, G2 = 25, G3 = 20). AUC = area under the curve. CI = confidence intervals.

	AUC	CI	*p*-Value	Sensibility	Specificity
TNFRII	0.970	(0.929–1.000)	8.0 × 10^−8^	100%	88%
TNFRII + MT-COX3	1.000	(1.000–1.000)	1.1 × 10^−8^	100%	100%
TNFRII + MT-ND1	1.000	(1.000–1.000)	1.1 × 10^−8^	100%	100%
TNFRII + MT-ATP8	0.998	(0.991–1.000)	1.2 × 10^−8^	100%	96%
TNFRII + MT-ATP6	1.000	(1.000–1.000)	1.1 × 10^−8^	100%	100%
TNFRII + MT-RNR1	0.970	(0.929–1.000)	8.0 × 10^−8^	100%	88%

**Table 6:** Results of receiver operating characteristic (ROC) curve and multivariate logistic analyses for predicting, respectively, KIM-1 alone or in combination with specific mitochondrial RNAs (MT-COX3, MT-ND1, MT-ATP8, MT-ATP6, or MT-RNR1) as biomarkers of kidney function impairment in DKD (eGFR stage G3 versus G2). *n* = 69 (G1 = 24, G2 = 25, G3 = 20). AUC = area under the curve. CI = confidence intervals.

	AUC	CI	*p*-Value	Sensitivity	Specificity
KIM-1	0.818	(0.687–0.949)	2.8 × 10^−4^	80%	80%
KIM-1 + MT-COX3	0.918	(0.836–1.000)	2.0 × 10^−6^	85%	88%
KIM-1 + MT-ND1	0.914	(0.822–1.000)	2.0 × 10^−6^	90%	88%
KIM-1 + MT-ATP8	0.902	(0.811–0.993)	4.0 × 10^−6^	95%	80%
KIM-1 + MT-ATP6	0.926	(0.849–1.000)	1.0 × 10^−6^	95%	80%
KIM-1 + MT-RNR1	0.876	(0.777–0.975)	1.8 × 10^−5^	65%	96%

**Table 7:** Results of receiver operating characteristic (ROC) curve and multivariate logistic analyses for predicting, respectively, TNFR-1 alone or in combination with specific mitochondrial RNAs (MT-COX3, MT-ND1, MT-ATP8, or MT-ATP6) as biomarkers of kidney function impairment in DKD (eGFR stage G3 versus G2) in normoalbuminuric (upper panel) or microalbuminuric patients (lower panel). Normoalbuminuric patients *n* = 36 (G1 = 13, G2 = 13, G3 = 10); microalbuminuric patients *n* = 33 (G1 = 11, G2 = 12, G3 = 10). AUC: area under the curve, CI: confidence interval.

**Normoalbuminuric Patients**					
	**AUC**	**CI**	***p*** **-Value**	**Sensitivity**	**Specificity**
TNFRI	0.869	0.714–1.000	3.0 × 10^−3^	80%	92.3%
TNFRI + MT-COX3	0.969	0.909–1.000	1.6 × 10^−4^	100%	84.6%
TNFRI + MT-ND1	0.954	0.875–1.000	2.5 × 10^−4^	100%	84.6%
TNFRI + MT-ATP8	0.992	0.968–1.000	7.5 × 10^−5^	100%	92.3%
TNFRI + MT-ATP6	0.969	0.909–1.000	1.6 × 10^−4^	100%	92.3%
**Microalbuminuric Patients**					
	**AUC**	**CI**	***p*** **-Value**	**Sensitivity**	**Specificity**
TNFRI	0.967	0.902–1.000	2.2 × 10^−4^	100%	83.3%
TNFRI + MT-COX3	1.000	1.000–1.000	7.6 × 10^−5^	100%	100%
TNFRI + MT-ND1	1.000	1.000–1.000	7.6 × 10^−5^	100%	100%
TNFRI + MT-ATP8	1.000	1.000–1.000	7.6 × 10^−5^	100%	100%
TNFRI + MT-ATP6	1.000	1.000–1.000	7.6 × 10^−5^	100%	100%

**Table 8:** Results of receiver operating characteristic (ROC) curve and multivariate logistic analyses for predicting, respectively, TNFRII alone or in combination with specific mitochondrial RNAs (MT-COX3, MT-ND1, MT-ATP8, or MT-ATP6) as biomarkers of kidney function impairment in DKD (eGFR stage G3 versus G2) in normoalbuminuric patients. Normoalbuminuric patients *n* = 36 (G1 = 13, G2 = 13, G3 = 10). AUC: area under the curve, CI: confidence interval.

Normoalbuminuric Patients					
	AUC	CI	*p*-Value	Sensibility	Specificity
TNFRII	0.946	0.861–1.000	3.2 × 10^−5^	100%	76.9%
TNFRII + COX3	1.000	1.000–1.000	5.6 × 10^−5^	100%	100%
TNFRII + MTND1	1.000	1.000–1.000	5.6 × 10^−5^	100%	100%
TNFRII + ATP8	1.000	1.000–1.000	5.6 × 10^−5^	100%	100%
TNFRII + ATP6	1.000	1.000–1.000	5.6 × 10^−5^	100%	100%

**Table 9:** Results of receiver operating characteristics (ROC) curve and multivariate logistic analyses for predicting, respectively, KIM-1 alone or in combination with specific mitochondrial RNAs (MT-COX3, MT-ND1, MT-ATP8, or MT-ATP6) as biomarkers of kidney function impairment in DKD (eGFR stage G3 versus G2) in microalbuminuric patients *n* = 33 (G1 = 11, G2 = 12, G3 = 10). AUC: Area Under the Curve, CI: Confidence Interval.

Microalbuminuric Patients					
	AUC	CI	*p*-Value	Sensibility	Specificity
KIM-1	0.858	0.702–1.000	5.0 × 10^−3^	90%	75%
KIM-1 + MT-COX3	0.858	0.673–1.000	5.0 × 10^−3^	100%	83.3%
KIM-1 + MT-ND1	0.875	0.707–1.000	3.0 × 10^−3^	100%	83.3%
KIM-1 + MTATP8	0.858	0.702–1.000	5.0 × 10^−3^	90%	75%
KIM-1 + MTATP6	0.867	0.691–1.000	4.0 × 10^−3^	100%	83.3%

## Data Availability

Not applicable.

## Supplementary Materials

The supporting information can be downloaded at https://www.mdpi.com/article/10.3390/ijms24119412/s1.
