# Supplementary material for: Identification of a New RNA and Protein Integrated Biomarker Panel Associated with Kidney Function Impairment in DKD: Translational Implications
Source: Int J Mol Sci. 2023 May 28;24(11):9412. doi: 10.3390/ijms24119412 (PMC10253864; doi:10.3390/ijms24119412)
Supplement: Supplementary file 1 [file ijms-24-09412-s001.zip › ijms-2377850-supplementary.pdf]

Clinical Parameters	G1	G2	G3	p-Value
**Age**	67.30 ± 4.79	68.00 ± 5.10	70.60 ± 4.97	0.0825
**Gender (% M)**	78.26	80.77	75.00	0.8953
**BMI (kg/m2)**	27.25 (25.92–33.49)	29.17 (27.90–32.46)	29.76 (24.98–34.69)	0.6300
**WC (cm)**	104.10 ± 14.15	108.60 ± 12.97	107.50 ± 9.43	0.4900
**AST (UI/L)**	24.00 (22–29)	25.00 (21–34.50)	24.00 (21.50–30)	0.8400
**ALT (UI/L)**	31.00 ± (21–38)	25.50 ± (19–45)	18.50 ± (13–29.50)	0.0600
**COL. TOT (mg/dL)**	158.00 (122–180)	181.50 (143.5–207)	171.50 (141.3–209.5)	0.1700
**HDL (mg/dL)**	47.96 ± 9.38	45.65 ± 12.78	47.25 ± 11.20	0.7700
**LDL (mg/dL)**	75.40 (51.60–100.60)	96.70 (78.25–126.70)	77.40 (68.25–139.30)	0.0900
**Triglycerides (mg/dL)**	146.00 (83–202)	126.00 (88.75–244)	133.50 (108–156)	0.9200
**Uric acid (mg/dL)**	5.17 ± 1.63	6.25 ± 1.51	6.59 ± 1.25	0.0100
**BUN (mg/dL)**	18.22 (21.50–26.64)	21.96 (23.71–28.97)	33.41 (38.43–48.13)	<0.0001
**HBA1c (%)**	7.70 ± 1.34	7.70 ± 1.64	8.00 ± 1.35	0.9400
**Creatinine (mg/dL)**	0.73 (0.66–0.81)	0.96 (0.85–1.05)	1.40 (1.08–1.48)	<0.0001
**Albuminuria (mcg/mL)**	25.00 (12–33)	24.50 (6.75–124.8)	17.00 (10.50–37.75)	0.7900
**ALB/CREAT**	26.00 (14–45)	32.00 (8.5–72.5)	28.50 (13.25–89)	0.7900
**WBC (μL−1)**	7900 (5700–8900)	6200 (5200–8125)	7550 (5800–8875)	0.1400
**HCT (%)**	44.40 (42.20–46)	42.60 (39.93–45.13)	40.05 (37.60–41.70)	0.0100
**Systolic BP (mmHg)**	120.7 ± 11.20	125 ± 10.54	129.8 ± 11	0.0277
**Dyastolic BP (mmHG)**	75 ± 7.43	79.36 ± 6.90	80.65 ± 6.30	0.0210
**Hyperthesis subjects (% H)**	58.3	68	95	0.0208

Table S1. Demographic and clinical data of the validation cohort. WC: Waist Circumference; BUN: Blood Urea Nitrogen; HbA1c: Hemoglobin A1; WBC: White Blood Cells; HCT: Haematocrit; BP: Blood Pressure. Statistical p-value was evaluated through One-way ANOVA or Kruskal Wallis test respectively for parametric or non-parametric data.

	G1 (n=24)	G2 (n=25)	G3 (n=20)
**ACEi/ARBs – n (%)**	14 (58.3%)	17 (68%)	17 (85.0%)
**Other antihypertensive Drugs – n (%)**	12 (50.0%)	14 (56.0%)	15 (75.0%)
**Metformin – n (%)**	19 (79.2%)	16 (64.0%)	9 (45.0%)*
**TZD – n (%)**	0 (0%)	1 (4.0%)	0 (0%)
**Repaglinide – n (%)**	1 (4.2%)	0 (0%)	1 (5.0%)
**Sulphonylureas – n (%)**	0 (0%)	0 (0%)	3 (15.0%)*#
**GLP1-RAs – n (%)**	3 (12.5%)	1 (4.0%)	0 (0%)
**SGLT2i – n (%)**	6 (25.0%)	6 (24.0%)	7 (35.0%)
**DPP4i – n (%)**	1 (4.2%)	1 (4.0%)	3 (15.0%)
**Insuline – n (%)**	6 (25.0%)	12 (48.0%)	8 (40.0%)
**Antiplatelets drugs n (%)**	8 (33.3%)	12 (48.0%)	8 (40.0%)
**Statins – n (%)**	19 (79.2%)	10 (40.0%)*	12 (60.0%)
**Allopurynol – n (%)**	0 (0%)	1 (4.0%)	4 (20.0%)*#

Table S2. Pharmacological therapy according to eGFR groups. ACEi: Angiotensin Converting Enzyme inhibitors; ARBs: Angiotensin Receptor Blockers; TZD: tiazolidinedions; GLP1-RAs: Glucagon-Like Peptide 1 Receptor Agonists; SGLT2i: Sodium-Glucose Transporter 2 inhibitors; DPP4i: DiPeptidyl Peptidase 4 inhibitors. Chi-square test: *p-value <0.05 vs group 1. #p-value <0.05 vs group 2.

	Normoalbuminuric (n=36)	Microalbuminuric (n=33)
**ACEi/ARBs – n (%)**	25 (69.4%)	23 (69.7%)
**Other antihypertensive Drugs – n (%)**	22 (61.1%)	19 (57.6%)
**Metformin – n (%)**	25 (69.4%)	19 (57.6%)
**TZD – n (%)**	1 (2.8%)	0 (0%)
**Repaglinide – n (%)**	1 (2.8%)	1 (3.0%)
**Sulphonylureas – n (%)**	3 (8.3%)	0 (0%)
**GLP1-RAs – n (%)**	2 (5.6%)	2 (6.0%)
**SGLT2i – n (%)**	11 (30.5%)	8 (24.2%)
**DPP4i – n (%)**	4 (11.1%)	1 (3.0%)
**Insulin – n (%)**	12 (33.3%)	14 (42.4%)
**Antiplatelets drugs n (%)**	17 (47.2%)	11 (33.3%)
**Statins – n (%)**	23 (63.9%)	18 (54.5%)
**Allopurynol – n (%)**	3 (8.3%)	2 (6.0%)

Table S3. Pharmacological therapy according to normoalbuminuric/microalbuminuric subjects. ACEi: Angiotensin Converting Enzyme inhibitors; ARBs: Angiotensin Receptor Blockers; TZD: tiazolidinedions; GLP1-RAs: Glucagon-Like Peptide 1 Receptor Agonists; SGLT2i: Sodium-Glucose Transporter 2 inhibitors; DPP4i: DiPeptidyl Peptidase 4 inhibitors. Statistical significance was evaluated through the chi-square test.
